# Weak Association between Skin Autofluorescence Levels and Prediabetes with an ILERVAS Cross-Sectional Study

**DOI:** 10.3390/nu14051102

**Published:** 2022-03-05

**Authors:** Enric Sánchez, Mohsen Kerkeni, Marta Hernández, Ricard Gavaldà, Ferran Rius, Ariadna Sauret, Gerard Torres, Marcelino Bermúdez-López, Elvira Fernández, Eva Castro-Boqué, Francisco Purroy, Dídac Mauricio, Cristina Farràs-Sallés, Miquel Buti, Pere Godoy, Reinald Pamplona, Albert Lecube

**Affiliations:** 1 Endocrinology and Nutrition Department, Diabetes and Metabolism (ODIM) Research Group, University Hospital Arnau de Vilanova, Obesity, IRBLleida, University of Lleida, 25198 Lleida, Spain; esanchez@irblleida.cat (E.S.); mhernandezg.lleida.ics@gencat.cat (M.H.); frius.lleida.ics@gencat.cat (F.R.); ariadnags973@gmail.com (A.S.); 2 Laboratory of Research on Biologically Compatible Compounds, Faculty of Dental Medicine, University of Monastir, Monastir 5000, Tunisia; mohsen.kerkeni@yahoo.fr; 3 Amalfi Analytics, Polytechnic University of Catalonia, 08034 Barcelona, Spain; rgavalda@irblleida.cat; 4 Precision Medicine in Chronic Diseases Group, IRBLleida, 25198 Lleida, Spain; gtorres@gss.scs.es; 5 Vascular and Renal Translational Research Group, IRBLleida, 25198 Lleida, Spain; mbermudez@irblleida.cat (M.B.-L.); efernandez@irblleida.cat (E.F.); ecastro@irblleida.cat (E.C.-B.); 6 Red de Investigación Renal, Instituto de Salud Carlos III (RedinRen-ISCIII), 28029 Madrid, Spain; 7 Stroke Unit, Clinical Neurosciences Group, University Hospital Arnau de Vilanova, IRBLleida, University of Lleida, 25198 Lleida, Spain; fpurroygarcia@gmail.com (F.P.); pere.godoy@gencat.cat (P.G.); 8 Endocrinology and Nutrition Department, Hospital de la Santa Creu i Sant Pau, Institut de Recerca Biomèdica Sant Pau (IIB Sant Pau), 08025 Barcelona, Spain; didacmauricio@gmail.com; 9 Centro de Investigación Biomédica en Red de Diabetes y Enfermedades Metabólicas Asociadas (CIBERDEM), Instituto de Salud Carlos III (ISCIII), 28029 Madrid, Spain; 10 Applied Epidemiology Research Group, IRBLleida. Unitat de Suport a la Recerca Lleida, Fundació Institut Universitari per a la Recerca a l’Atenció Primària de Salut Jordi Gol i Gurina (IDIAPJGol), 25198 Lleida, Spain; cfarras.lleida.ics@gencat.cat (C.F.-S.); mbuti.lleida.ics@gencat.cat (M.B.); 11 Department of Experimental Medicine, IRBLleida, University of Lleida, 25198 Lleida, Spain; reinald.pamplona@mex.udl.cat

**Keywords:** advanced glycation end-products, glycosylated hemoglobin, prediabetes, skin autofluorescence

## Abstract

A large body of evidence demonstrates a relationship between hyperglycemia and increased concentrations of advanced glycation end-products (AGEs). However, there is little information about subcutaneous AGE accumulation in subjects with prediabetes, and whether or not this measurement could assist in the diagnosis of prediabetes is unclear. A cross-sectional study was conducted in 4181 middle-aged subjects without diabetes. Prediabetes (*n* = 1444) was defined as a glycosylated hemoglobin (HbA1c) level between 39 and 47 mmol/mol (5.7 to 6.4%), and skin autofluorescence (SAF) measurement was performed to assess AGEs. A multivariable logistic regression model and receiver operating characteristic curve were used. The cohort consisted of 50.1% women with an age of 57 [52;62] years, a BMI of 28.3 [25.4;31.6] kg/m^2^, and a prevalence of prediabetes of 34.5%. Participants with prediabetes showed higher SAF than control participants (2.0 [1.7;2.2] vs. 1.9 [1.7;2.2], *p* < 0.001). However, HbA1c was not significantly correlated with SAF levels (r = 0.026, *p* = 0.090). In addition, the SAF level was not independently associated with prediabetes (OR = 1.12 (0.96 to 1.30)). Finally, there was no good cutoff point for SAF to identify patients with prediabetes (AUC = 0.52 (0.50 to 0.54), sensitivity = 0.61, and 1-specificity = 0.56). Given all of this evidence, we can conclude that although there is an increase in SAF levels in participants with prediabetes, the applicability and clinical relevance of the results is low in this population.

## 1. Introduction 

Advanced glycation end-products (AGEs) constitute a complex group of compounds formed by the slow non-enzymatic glycation of proteins, lipids, and nucleic acids, of which about 20 have been identified to date [1]. AGEs trigger the formation of cross-links between extracellular matrix basement membrane molecules and the activation of receptors for AGEs. These pathological mechanisms contribute to, among other issues, endothelial dysfunction, formation of reactive oxygen species, and vascular stiffness [2]. Aging leads to an increase in the concentration of AGEs in tissues and the circulation, which is accelerated by chronic hyperglycemia and smoking, resulting in increased oxidative stress and cellular damage [3,4]. New studies have suggested that the abundance of prooxidant AGEs in the highly industrialized food environment may explain the onset and progression of prediabetes to type 2 diabetes [5].

Prediabetes, an intermediate metabolic state between type 2 diabetes and normal glucose metabolism, showed a prevalence of 38.6% based on fasting plasma glucose (FPG)/hemoglobin A1c (HbA1c) in the adult population according to the National Health and Nutrition Examination Survey (NHANES) performed between 2017 and 2020 [6]. It is essential to note that prediabetes is not an inconsequential health condition, as it has been associated with an increased incidence of cardiovascular disease and diabetic microangiopathy compared with the population with normal glucose metabolism [7,8,9]. Subcutaneous AGE content, assessed by skin autofluorescence (SAF), has been implicated in the early development of both plaque-burden-associated risk factors and classic microangiopathic complications in diabetes [10,11,12]

It is well known that the main challenge in the prevention of type 2 diabetes is the early identification of individuals with prediabetes to introduce actions to postpone the onset of future disease [13]. However, the best way to perform this screening has yet to be decided. Thus, measures such as FPG, 2 h plasma glucose, and HbA1c are equally suitable for identifying prediabetes and diabetes, but not all of them identify the disease in the same individual [14]. The measurement of AGEs seems to be a suitable way to assess the effects of years of chronic non-diabetic hyperglycemia, and subcutaneous AGE deposition has been previously evaluated in populations at risk for diabetes with positive results [15,16,17,18,19,20]. However, it remains to be elucidated whether the measurement of AGE deposition in subcutaneous tissue can help to differentiate subjects with prediabetes from those with normal glucose metabolism. Therefore, the aim of our study was to evaluate the concentration of subcutaneous AGE in prediabetes, as well as determine whether its measurement could be a good option for the diagnosis of this metabolic state, by examining a population with low-to-moderate cardiovascular risk from the ILERVAS study.

## 2. Materials and Methods

### 2.1. Ethical Statement

The protocol was accepted by the ethics committee of the University Hospital Arnau de Vilanova (CEIC-1410). The clinical trial was conducted in accordance with the ethical guidelines of the Declaration of Helsinki and Spanish legislation on personal data protection. All subjects provided written informed consent.

### 2.2. Design of the Study and Data of the Study Participants

**Figure 1:**
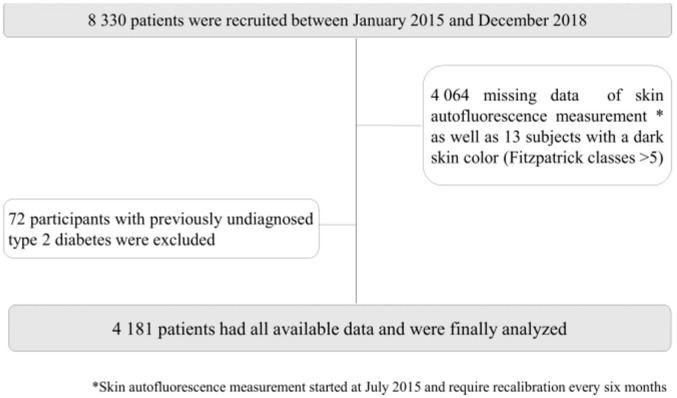
Flow diagram for the study population.

The ILERVAS project is an ongoing randomized intervention study with the aim of reducing cardiovascular events in Catalonia, Spain (ClinTrials.gov Identifier: NCT03228459) [21]. Between January 2015 and December 2018, 8330 middle-aged participants were recruited from various primary care centers. Inclusion criteria were women aged 50 to 70 years, men aged 45 to 65 years, and the presence of at least one cardiovascular risk factor (dyslipidemia, hypertension, obesity, smoking, and/or having a first-degree relative with premature cardiovascular disease). Exclusion criteria were a clinical history of cardiovascular disease, any type of diabetes, renal disease, active neoplasia, a life expectancy of less than 1 year, institutionalized population, and pregnancy. The study flow chart is displayed in Figure 1.

### 2.3. Measurement of Skin Autofluorescence 

SAF was calculated using the AGE Reader™ (DiagnOptics Technologies, Groningen, The Netherlands), a fully automated non-invasive tool that assesses AGE deposition on the forearm using ultraviolet A light [22]. The mean value of three analyses (arbitrary units: AUs) was recorded. Areas of tattooed or cosmetically colored skin and areas with many freckles or vessels close to the skin surface were avoided. A single device, maintained and calibrated by the manufacturer according to their recommendations, was used for all measurements. Repeated SAF measurements with the AGE Reader™ device taken on a single day showed an overall Altman error rate of 5.03%, and intra-individual seasonal variance showed an Altman error rate of 5.87% [22]. 

### 2.4. Diagnosis of Prediabetes and Type 2 Diabetes

The current American Diabetes Association guidelines define type 2 diabetes as HbA1c ≥ 48 mmol/mol (≥6.5%), prediabetes as HbA1c between 39 and 47 mmol/mol (5.7 to 6.4%), and normal glucose metabolism as HbA1c < 39 mmol/mol (<5.7%) [14]. A total of 72 participants with previously undiagnosed type 2 diabetes were excluded from the analysis, which was finally performed in 4181 individuals. The HbA1c test was performed on capillary blood using a point-of-care device (Cobas B 101^®^, Roche Diagnostics S.L., Sant Cugat del Vallès, Spain) based on a latex agglutination inhibition immunoassay technique that meets generally accepted performance standards for HbA1c. Subjects with prediabetes were divided into those with low (5.7% to 6.0%) and high (6.1% to 6.4%) HbA1c due to the non-normal distribution of the variable. Body weight and height were measured without shoes and with little clothing, and body mass index (BMI) was determined by dividing weight in kilograms by height in meters squared. Smoking status (never, former, or current smoker) was also obtained. Smokers who quit smoking one year or more before the visit were considered ex-smokers.

### 2.5. Statistical Analysis

The normal distribution of the variables was assessed using the Shapiro–Wilk test, and the data are expressed as median (interquartile range) or as a percentage. The Mann–Whitney U test was used to compare continuous variables, while Pearson’s chi-square test was used to compare categorical data. The relationship between continuous variables was examined using Spearman’s correlation test.

Multivariable logistic regression was performed to identify variables that are independently related to prediabetes. Variables with a potential impact on the measurement of prediabetes (i.e., age, sex, BMI, and smoking) and the levels of SAF were entered into the model. Additionally, the multivariable logistic regression model was calibrated by both the Hosmer–Lemeshow test of fit and area under the ROC curve.

Finally, the area under the receiver operating characteristic (ROC) curve showing sensitivity/specificity was calculated to examine the diagnostic ability of skin autofluorescence to discriminate prediabetes. The total area under the ROC curve was interpreted using the following guidelines: 0.9–1.0, excellent; 0.8–0.9, good; 0.7–0.8, fair; 0.6–0.7, poor; and 0.5–0.6, not useful. All *p*-values were based on a two-sided statistical significance test. Significance was set at the level of *p* < 0.050. All statistical investigations were performed with the SSPS statistical package (IBM SPSS Statistics for Windows, version 27.0. Armonk, NY, USA).

## 3. Results 

The main clinical characteristics of the ILERVAS population according to the presence or absence of prediabetes are shown in Table 1. Subjects with prediabetes were older, mainly women, and had a higher proportion of non-communicable diseases than controls. Subjects with prediabetes presenting high HbA1c values (6.1% to 6.4%) appeared to be under-represented (17.5%).

**Figure 2:**
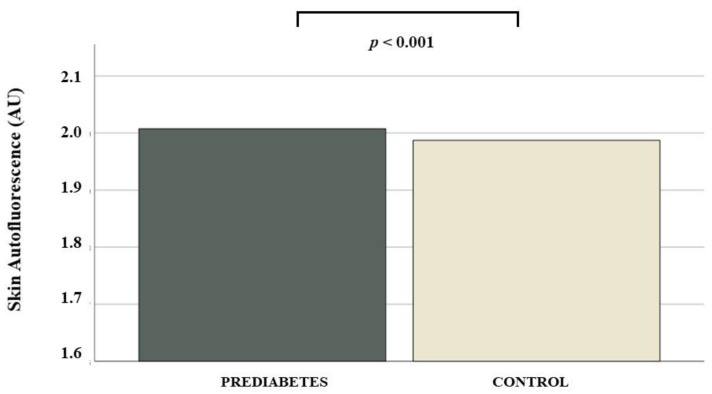
Plot displaying the skin autofluorescence value according to the presence prediabetes.

Participants with prediabetes showed small but statistically significant increases in SAF compared to controls (2.0 [1.7;2.2] vs. 1.9 [1.7;2.2], *p* < 0.001) (Figure 2). Furthermore, when this comparison was made in patients with prediabetes, similar results were obtained when comparing those with higher HbA1c levels (6.1% to 6.4%) with those with a lower HbA1c concentration for SAF levels: 2.0 [1.7;2.2] vs. 1.9 [1.7;2.2], *p* < 0.001. 

According to the quartiles of SAF levels, the prevalence of prediabetes increased significantly (31.0%, 35.7%, 37.9%, and 34.8%, respectively, *p* = 0.006). Similar results were observed when HbA1c was evaluated according to SAF quartiles (*p* = 0.005). In the whole population, the SAF level was not significantly correlated with HbA1c (r = 0.026, *p* = 0.090). 

Furthermore, the multivariable logistic regression model (Table 2) showed that male sex and smoking, but not the SAF level, were significantly and independently associated with prediabetes. The results of this model were similar after stratifying for obesity, hypertension, and dyslipidemia.

**Figure 3:**
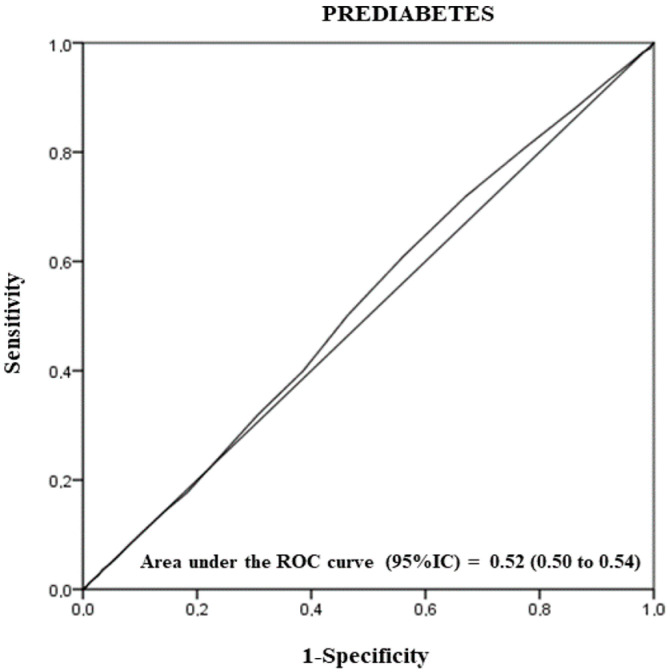
Receiver operating characteristic (ROC) curve analysis to evaluate the accuracy of skin autofluorescence in distinguishing prediabetes from cases with normal metabolism, together with sensitivity/specificity data.

Finally, ROC analysis revealed that SAF measurement was not useful (considering both sensitivity and specificity) for identifying patients with prediabetes in our population (AUC = 0.52 (0.50 to 0.54), sensitivity = 0.61, and 1-specificity = 0.56) (Figure 3).

## 4. Discussion

In the present study, we provide evidence that, although the prediabetes stage is associated with a modest increase in SAF, its measurement does not appear to be useful for screening for prediabetes, at least not in the population with low-to-moderate cardiovascular risk from the ILERVAS study. The period of mild hyperglycemia preceding the development of type 2 diabetes exerts a negative effect on health, facilitating the development of various risk factors associated with cardiovascular disease and classic microangiopathic complications [8,9]. The role of AGEs in the etiopathogenesis of late complications in type 2 diabetes is well known. In addition, a recent study suggested that SAF is associated with vascular stiffness in prediabetes and normoglycemia in a population-based cohort without high CV disease risk [23].

In recent years, much research has been conducted to explain the negative impact of AGEs on human health and to identify the pathological mechanisms involved in the development and progression of type 2 diabetes, such as insulin resistance and cellular dysfunction [24]. The association of AGEs with HbA1c and chronic complications of type 2 diabetes has also been described, suggesting that the measurement of AGEs could be a potential biomarker to predict progression from normal glucose metabolism to type 2 diabetes [25,26,27,28]. For this reason, the measurement of SAF, using increasingly smaller, more manageable, and less expensive tabletop devices, is becoming more widespread in the assessment of cardiovascular risk [29]. It could also offer an opportunity for prediabetes screening in populations with varying degrees of CV risk. However, our study failed to establish a correlation between SAF and metabolic control in the prediabetes phase. We did find a higher concentration of AGEs in subjects with prediabetes compared to those with normal glucose metabolism. However, although this difference was statistically significant, probably due to the large population evaluated, it does not appear to be clinically relevant. 

Among individuals at high risk for type 2 diabetes, SAF has previously been shown to have equal or superior diagnostic performance for prediabetes compared to FPG and HbA1c [15,16,17,18,19,20,30]. In the study by Maynard et al. involving 351 subjects with one or more diabetes risk factors, the sensitivity differential indicated that SAF was able to identify 28.8% more individuals with 2-h OGTT values ≥140 mg/dL than FPG testing and 17.1% more than HbA1c testing [16]. Similarly, in the study by Smit et al. in 218 subjects meeting at least one metabolic syndrome criterion, the performance of a SAF-based decision model was better than conventional FPG and comparable to HbA1c in detecting impaired glucose tolerance or diabetes [15]. Furthermore, in both the NSEEDS and ENGINE studies, SAF was compared with FPG and HbA1c for the detection of abnormal glucose tolerance in subjects at risk for type 2 diabetes but without an established diagnosis [18,19]. Finally, 1 h post–glucose load plus SAF appeared to be the best combination in the assessment of prediabetes among the Diabetes Prediction and Screening Observational (DIAPASON) study cohort, which included subjects aged 40 to 75 years and a FINDRISC ≥9, [17]. However, in our study, although we showed slightly elevated levels of SAF in the ILERVAS population when prediabetes was defined according to HbA1c criteria, we did not observe this clinical utility

This study has some limitations. First, it would have been interesting to compare the SAF data with plasma AGE concentrations. However, AGE deposition seems to be a more reliable measure of the negative impact of these compounds on human health since SAF remains stable for a long time due to its slow turnover. Second, our population had an HbA1c level of 5.8%, which is in the lower cutoff range for the diagnosis of prediabetes. This would suggest that subjects with higher HbA1c levels within the prediabetes range were under-represented. SAF is related to metabolic control about 10 years earlier and not to current metabolic control, which may explain why SAF shows a low discriminatory ability compared to HbA1c, which captures more recent effects in its measurement [31]. Finally, in contrast to previous studies, we did not evaluate a population at high risk of developing diabetes but a population at low-to-moderate cardiovascular risk. This difference between study populations could lead to different results. 

## 5. Conclusions

In sum, there is a modest increase in SAF levels in participants with prediabetes that is mainly associated with male sex and smoking. However, we did not observe a significant and independent association between the presence of prediabetes and high SAF levels. In addition, there is not a cutoff point for SAF that has both the sensitivity and specificity needed to identify patients with prediabetes. Therefore, SAF does not appear to be a good tool to identify subjects with prediabetes among the population with low-to-moderate moderate CV risk.

## Figures and Tables

**Table 1:** Main clinical characteristics of the study population according to glucose abnormalities.

	Prediabetes (*n* = 1444)	Control Group (*n* = 2737)
Age (years)	59 [54; 64]	56 [52; 62]
Women, *n* (%)	827 (57.3)	1268 (46.3)
BMI (kg/m^2^)	29.5 [26.7; 33.1]	27.7 [24.8; 30.8]
HbA1c (mmol/mol)	40 [39; 42]	36 [33; 37]
HbA1c (%)	5.8 [5.7; 6.0]	5.4 [5.2; 5.5]
Hypertension, *n* (%)	684 (47.4)	992 (36.2)
Dyslipidemia	865 (59.9)	1363 (49.8)
Obesity, *n* (%)	581 (40.2)	758 (27.7)
Current or former smoker, *n* (%)	828 (57.3)	1819 (66.5)

Data are presented as a median [interquartile range] or *n* (percentage). HbA1c: glycated hemoglobin; BMI: body mass index.

**Table 2:** The multivariable logistic regression model for presence of prediabetes.

Prediabetes		OR (95% CI)	*p* Value
Sex	Women	Reference	
	Men	1.34 (1.16 to 1.55)	<0.001
Age (years)		1.04 (1.03 to 1.05)	<0.001
Body mass index (kg/m^2^)		1.09 (1.07 to 1.10)	<0.001
Smoking status	Never	Reference	
	Current or former	1.08 (0.93 to 1.25)	0.326
Skin autofluorescence (AU)		0.97 (1.00 to 0.85)	0.969
Hosmer–Lemeshow test of fit		0.65 (0.64 to 0.67)	0.002 <0.001
Area under the ROC curve			

## Data Availability

The data presented in this study are available on request from the corresponding author. The data are not publicly available due to the signed consent agreements around data sharing, which only allow access to the researchers of the study following the project purposes. Requestors wishing to access the data used in this study can make a request to A.L. and M.B.-L. The request will be subjected to approval and formal agreements regarding confidentiality and secure data storage being signed the data would be the provided.
